# Author Correction: Intensification of terrestrial carbon cycle related to El Niño–Southern Oscillation under greenhouse warming

**DOI:** 10.1038/s41467-017-02461-9

**Published:** 2018-01-15

**Authors:** Jin-Soo Kim, Jong-Seong Kug, Su-Jong Jeong

**Affiliations:** 1 Division of Environmental Science and Engineering, Pohang University of Science and Technology (POSTECH), Pohang, 37673 South Korea; 2 School of Environmental Science and Engineering, Southern University of Science and Technology (SUSTECH), Shenzhen, 518055 China

Correction to: *Nature Communications* 10.1038/s41467-017-01831-7, Article published online 22 November 2017

In the original version of this Article, the affiliation for Su-Jon Jeong was incorrectly given as ‘Southern University of Science and Technology of China (SUSTECH)’, instead of ‘Southern University of Science and Technology (SUSTECH)’. This has now been corrected in both the PDF and HTML versions of the Article.

