# Supplementary material for: Intensification of terrestrial carbon cycle related to El Niño–Southern Oscillation under greenhouse warming
Source: Nat Commun. 2017 Nov 22;8:1674. doi: 10.1038/s41467-017-01831-7 (PMC5698330; doi:10.1038/s41467-017-01831-7)
Supplement: Supplementary file 2 — Supplementary Information [file 41467_2017_1831_MOESM2_ESM.pdf]

Supplementary Table 1 | CMIP5 ESMs used in this study

Model Name	Modeling Center (or Group)
CESM1-CAM5	Community Earth System Model Contributors
CanESM2	Canadian Centre for Climate Modelling and Analysis
GISS-E2-H	NASA Goddard Institute for Space Studies
HadGEM2-ES	Met Office Hadley Centre
IPSL-CM5A-MR	Institute Pierre Simon Laplace
MIROC-ESM	Japan Agency for Marine-Earth Science and Technology, Atmosphere and Ocean Research Institute (The University of Tokyo), and National Institute for Environmental Studies
MPI-ESM-LR	Max Planck Institute for Meteorology
NorESM1-M	Norwegian Climate Centre
bcc-csm1-1	Beijing Climate Center,<br>China Meteorological Administration

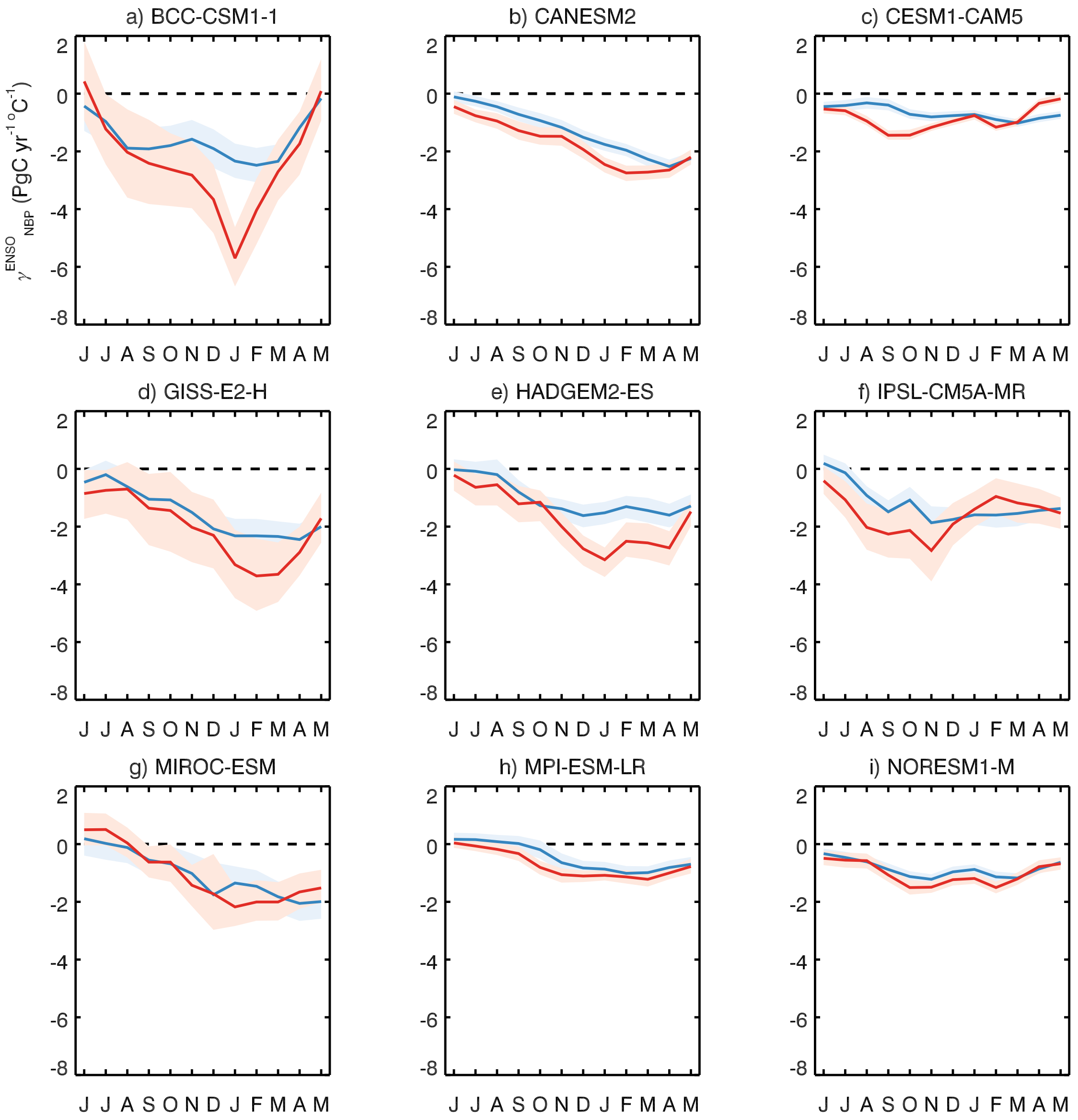

Supplementary Figure 1 | Net biome production sensitivities to ENSO in individual models. Regression coefficients of carbon flux anomalies in 40°S–40°N on the December–February Niño3.4 index for net biome production based on the pre-industrial experiment (blue) and ECP4.5 (red). Shaded area indicates 95% confidence levels calculated using bootstrap method.

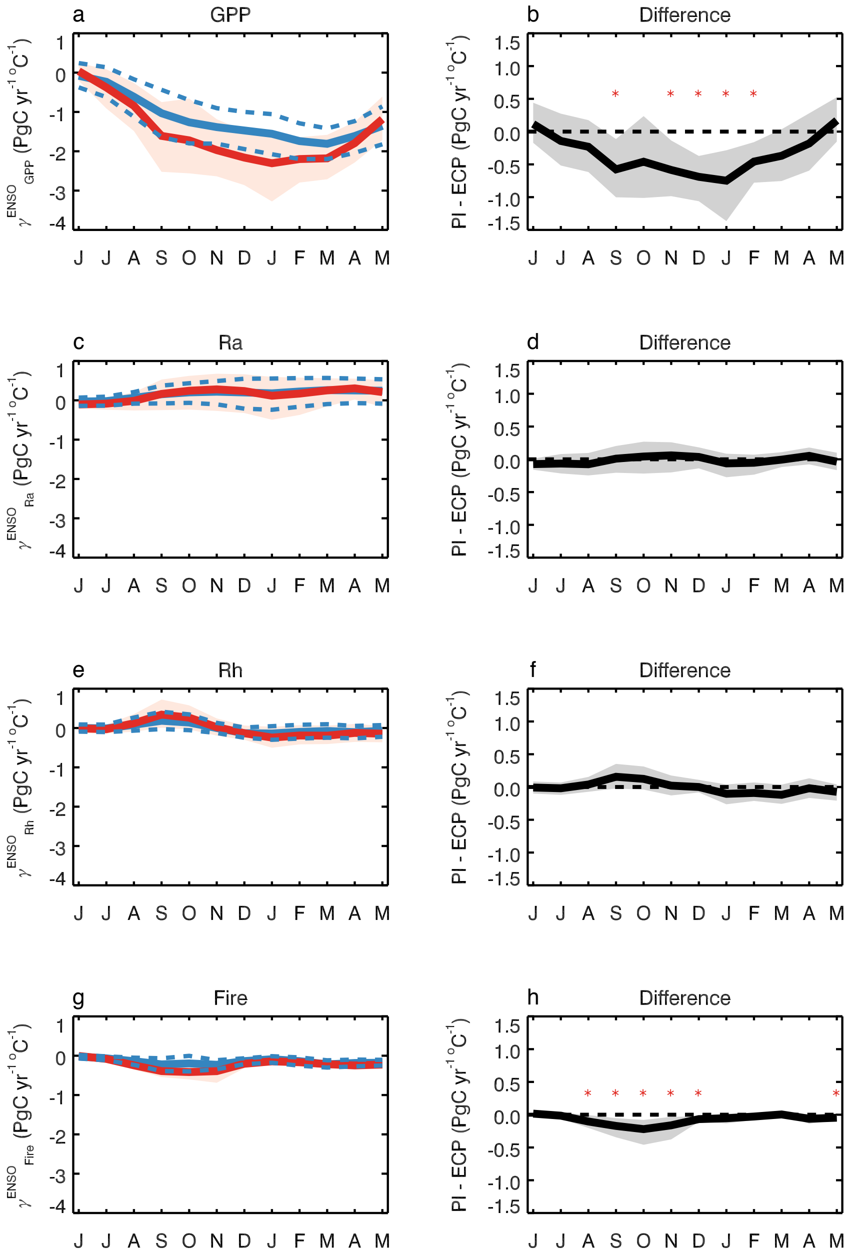

Supplementary Figure 2 | Terrestrial carbon flux sensitivities to ENSO. Regression coefficient of carbon flux anomalies in 40°S–40°N on the December–February Niño3.4 index for (**a**) gross primary production, (**c**) autotrophic respiration (**e**) heterotrophic respiration and (**g**) fire in the pre-industrial experiment (blue) and ECP4.5 (red) and the (**b**, **d**, **f**, **h**) difference between two experiments for multi-model ensemble (thick line) and 95% confidence level (shaded) based on bootstrap estimates.

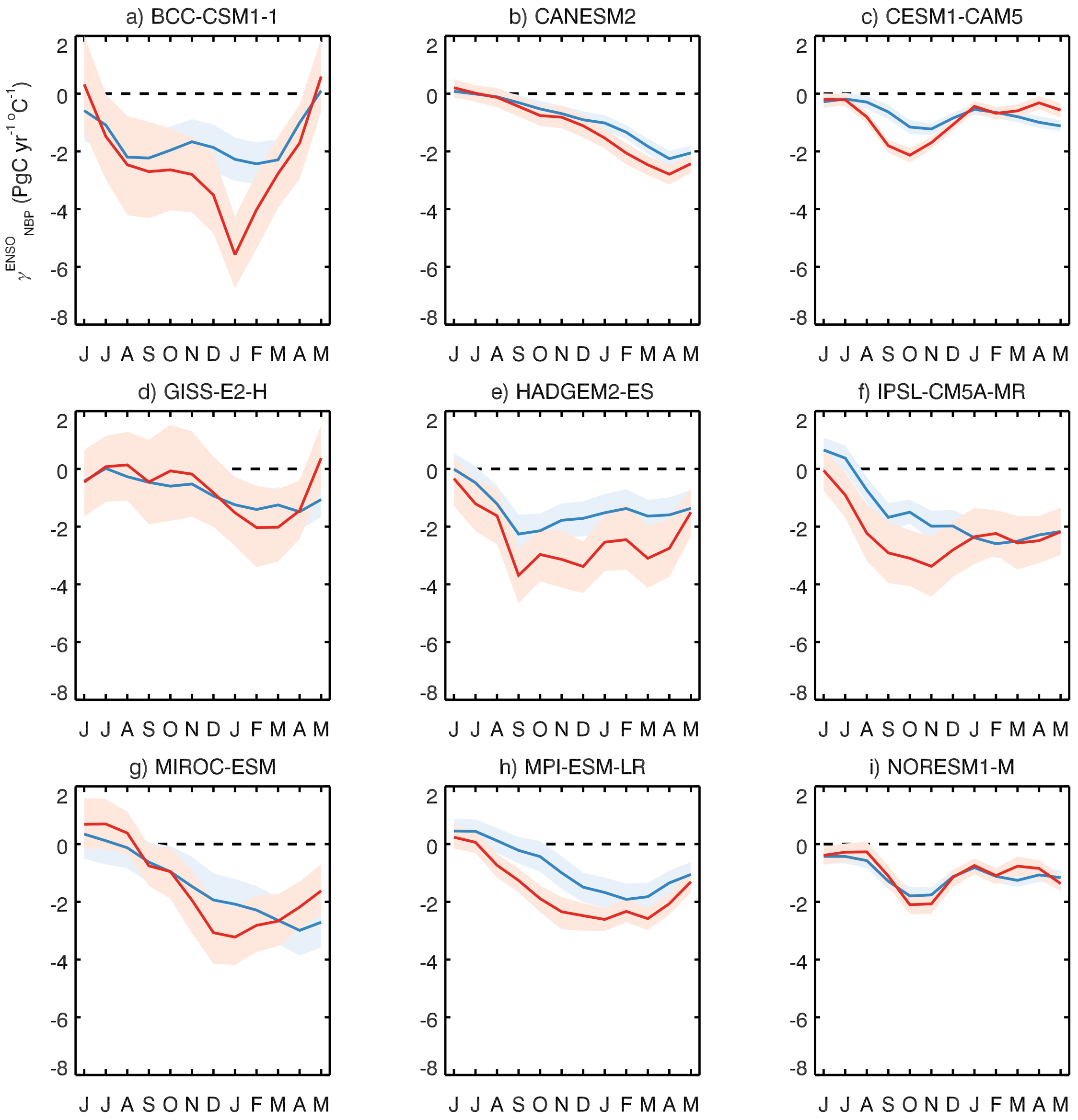

Supplementary Figure 3 | Gross primary production sensitivities to ENSO in individual models. Regression coefficients of carbon flux anomalies in 40°S–40°N on the December–February Niño3.4 index for gross primary production based on the pre-industrial experiment (blue) and ECP4.5 (red). Shaded area indicates 95% confidence levels calculated using bootstrap method.

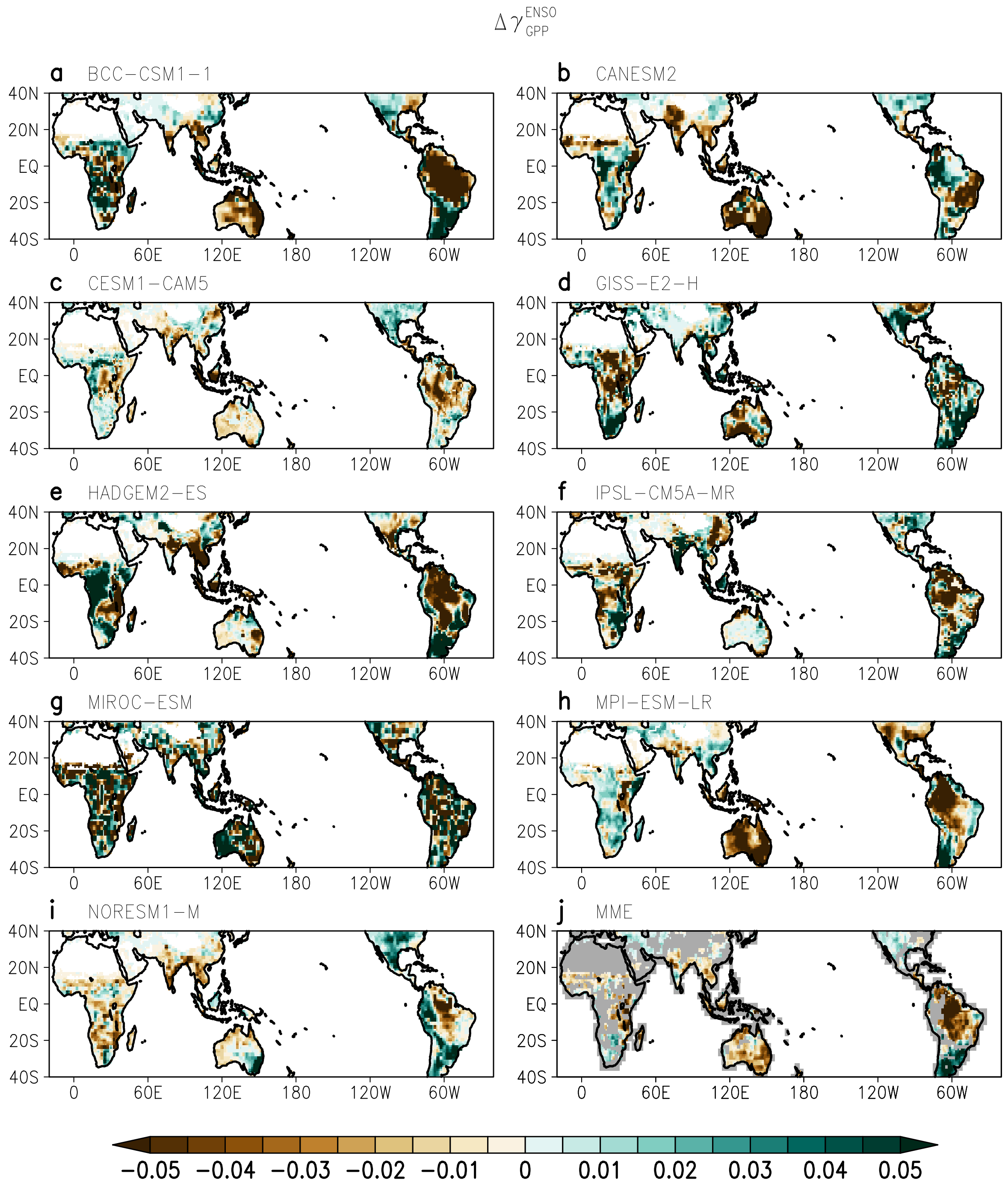

Supplementary Figure 4 | Changes in gross primary production response to ENSO ($\Delta\gamma_{GPP}^{ENSO}$). (a–i) Differences in the regression coefficient of gross primary production (GPP) anomalies during September to the following February on the DJF Niño3.4 index between the pre-industrial experiment and ECP4.5 for individual models. (**j**) Multi-model ensemble result

for changes in GPP response to ENSO. Gray area indicates non-significant region at 95% confidence level calculated using bootstrap method.

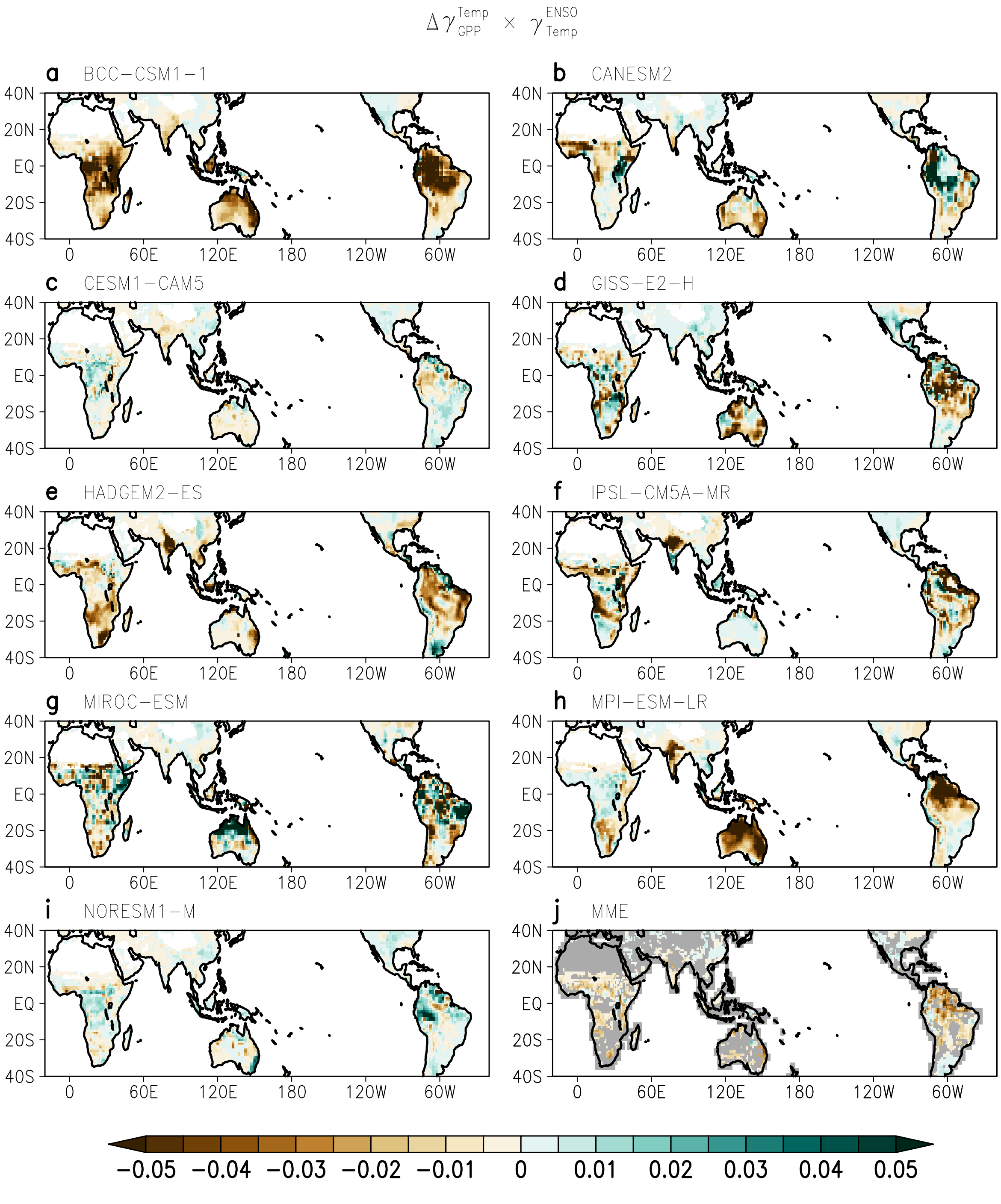

Supplementary Figure 5 | Contribution of the changes in the gross primary production sensitivity to temperature ($\Delta\gamma_{GPP}^{Temp}\gamma_{Temp}^{ENSO}$) (a–i) $\Delta\gamma_{GPP}^{Temp}\gamma_{Temp}^{ENSO}$ for individual models. **(j)** Multi-model ensemble result for $\Delta\gamma_{GPP}^{Temp}\gamma_{Temp}^{ENSO}$. Gray area indicates non-significant region at 95% confidence level calculated using bootstrap method.

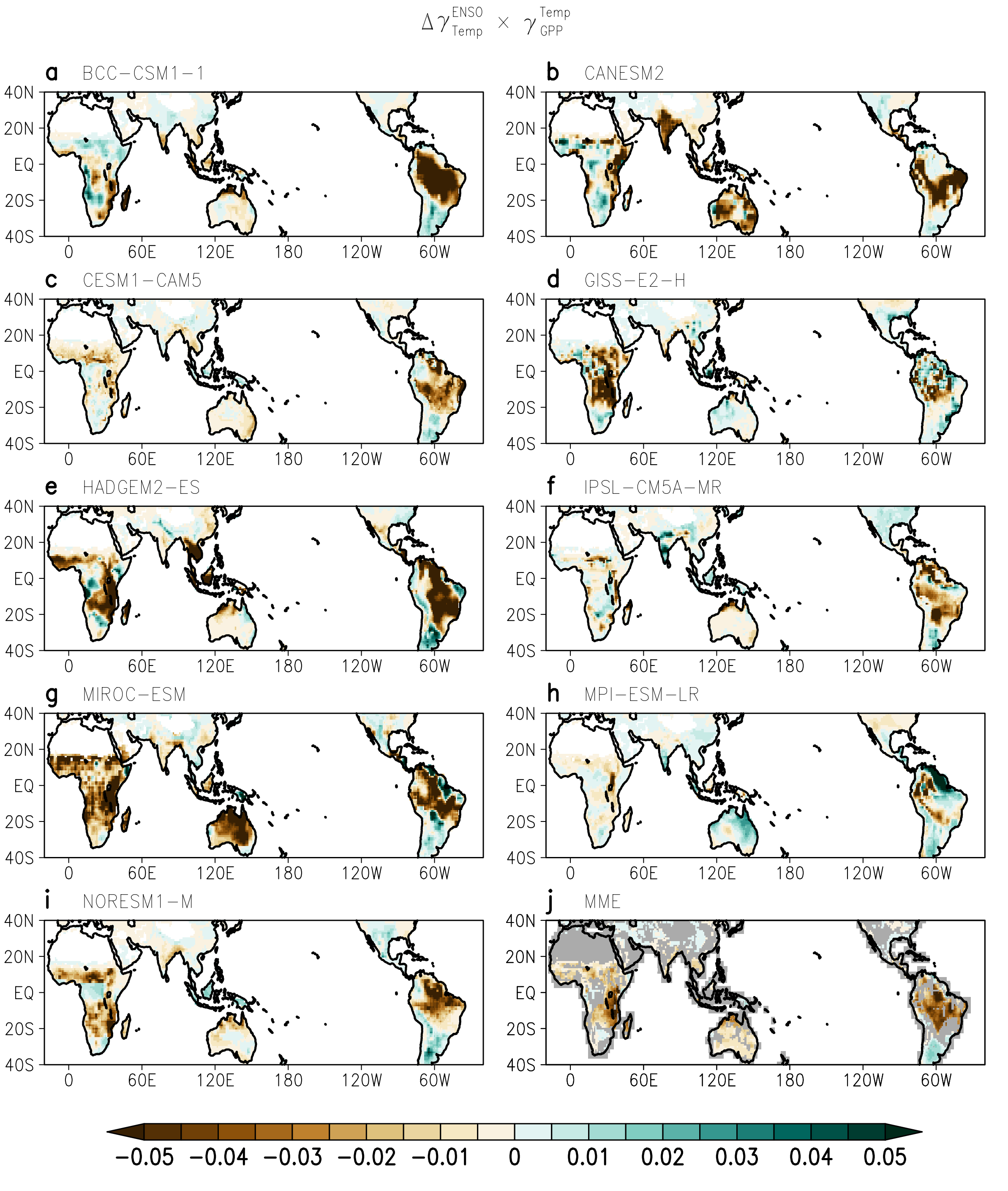

Supplementary Figure 6 | Contribution of the changes in the temperature response to ENSO ($\Delta\gamma_{Temp}^{ENSO}\gamma_{GPP}^{Temp}$) (a–i) $\Delta\gamma_{Temp}^{ENSO}\gamma_{GPP}^{Temp}$ for individual models. **(j)** Multi-model ensemble result for $\Delta\gamma_{Temp}^{ENSO}\gamma_{GPP}^{Temp}$. Gray area indicates non-significant region at 95% confidence level calculated using bootstrap method.

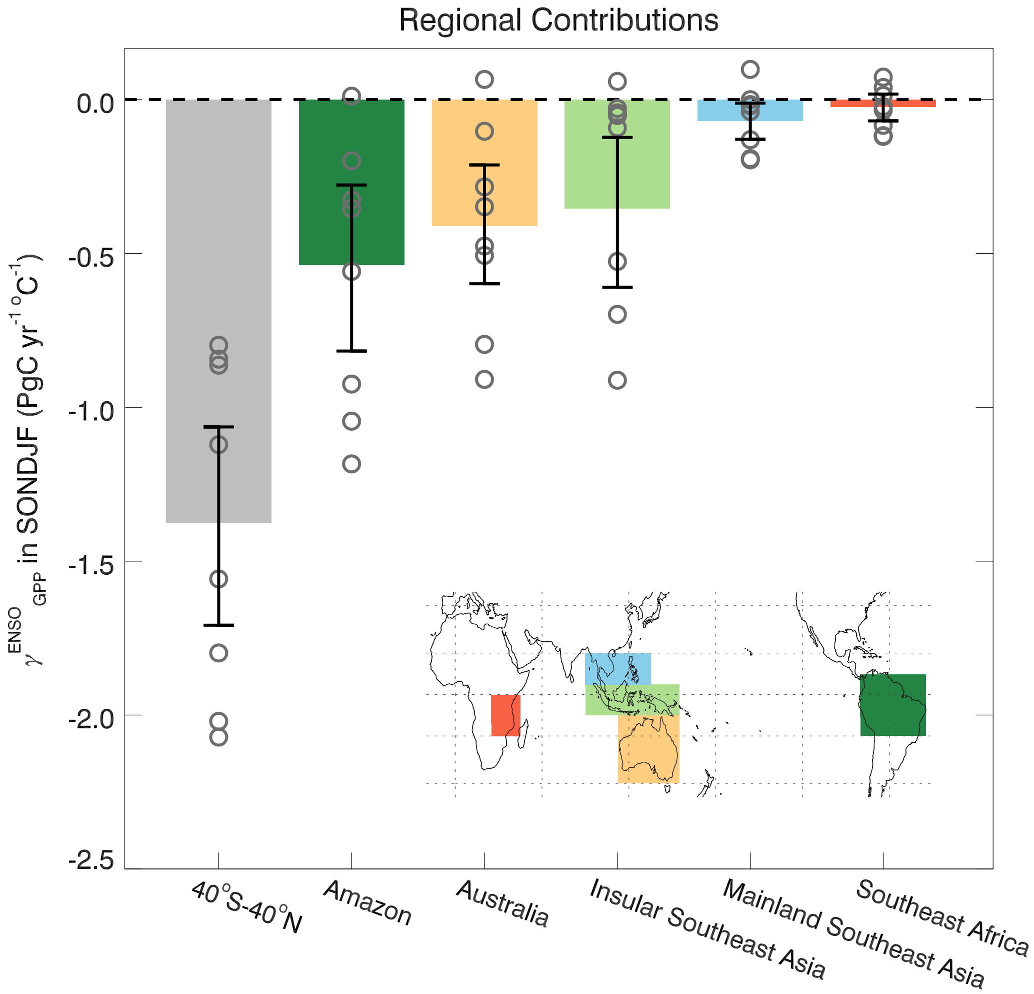

Supplementary Figure 7 | Regional contributions to the gross primary production response to ENSO. Regression coefficient of regional gross primary production anomalies during September to the following February on December–February Niño3.4 index in the pre-industrial experiment. Each bar shows the Multi-model ensemble results based on each region, as marked on the map. Open circles denote individual Earth System Model results. Error bars indicate 95% confidence levels based on bootstrap estimation.

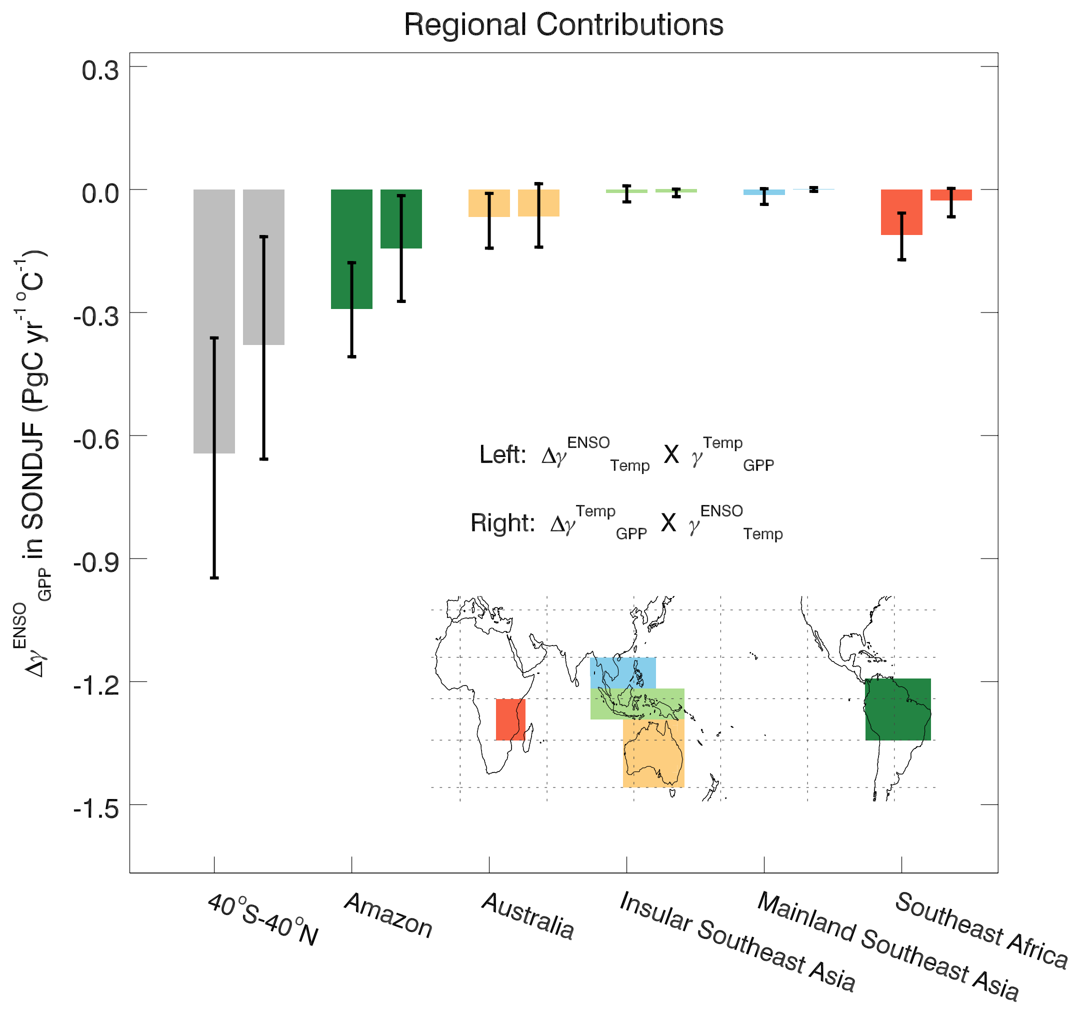

Supplementary Figure 8 | Regional contribution in Fig 2c,e. Regional contribution of the changes the surface temperature response to ENSO ($\Delta\gamma_{\mathrm{Temp}}^{\mathrm{ENSO}}\gamma_{\mathrm{GPP}}^{\mathrm{Temp}}$; left bars) and gross primary production sensitivity to temperature ($\Delta\gamma_{\mathrm{GPP}}^{\mathrm{Temp}}\gamma_{\mathrm{Temp}}^{\mathrm{ENSO}}$; right bars). Each bar shows the multi-model ensemble results based on each region, as marked on the map. Error bars indicate 95% confidence levels based on bootstrap estimation.

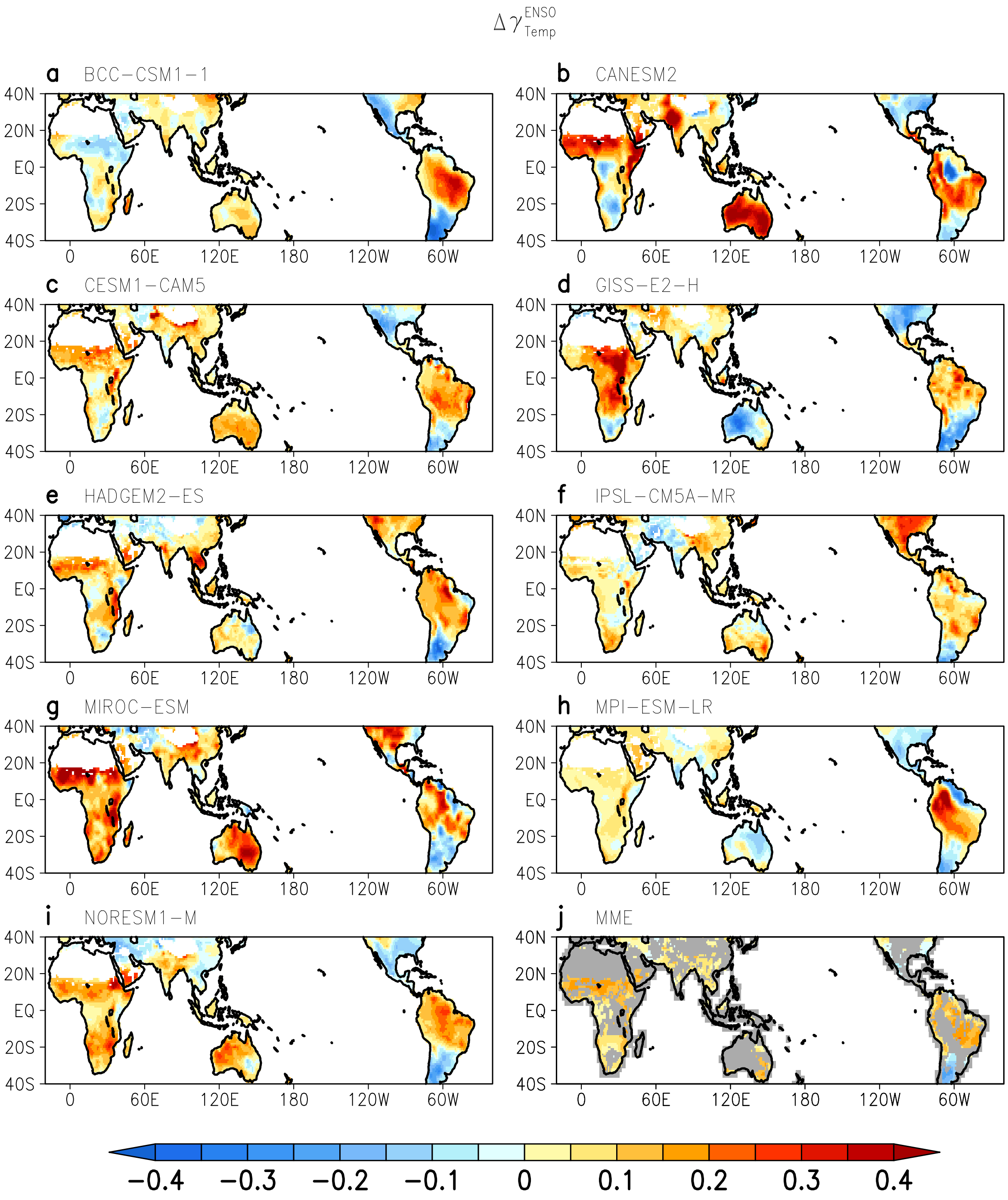

Supplementary Figure 9 | Future changes of temperature sensitivities to ENSO ($\Delta\gamma^{\mathrm{ENSO}}_{\mathrm{Temp}}$). **(a–i)** Regression coefficient differences of the local surface temperature anomalies during September to the following February on the December–February Niño3.4 index between the pre-industrial experiment and ECP4.5 for individual models. **(j)** Multi-model

ensemble result for $\Delta\gamma_{\mathrm{Temp}}^{\mathrm{ENSO}}$. Gray area indicates non-significant region at 95% confidence level calculated using bootstrap method.

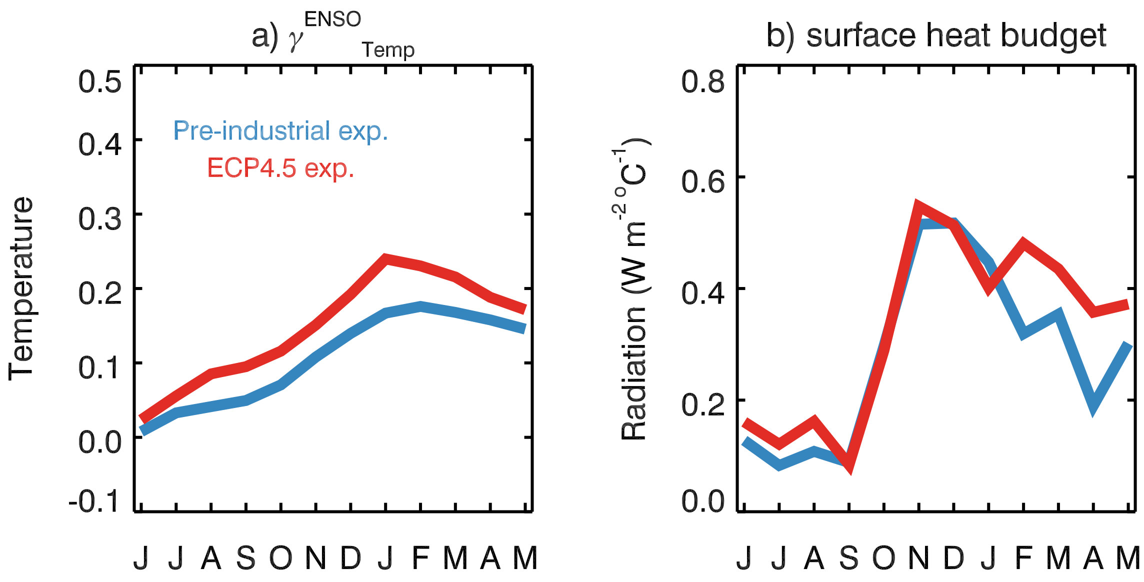

Supplementary Figure 10 | γ^{ENSO}_{Temp} and total summation of radiation in 40°S–40°N. (a) Multi-model ensemble γ^{ENSO}_{Temp} for pre-industrial (blue) and ECP4.5 (red) scenario. (b) total summation of radiation that downward/upward long/short wave radiation and sensible/latent heat in the pre-industrial experiment and ECP4.5 (W m^{-2} °C^{-1}).

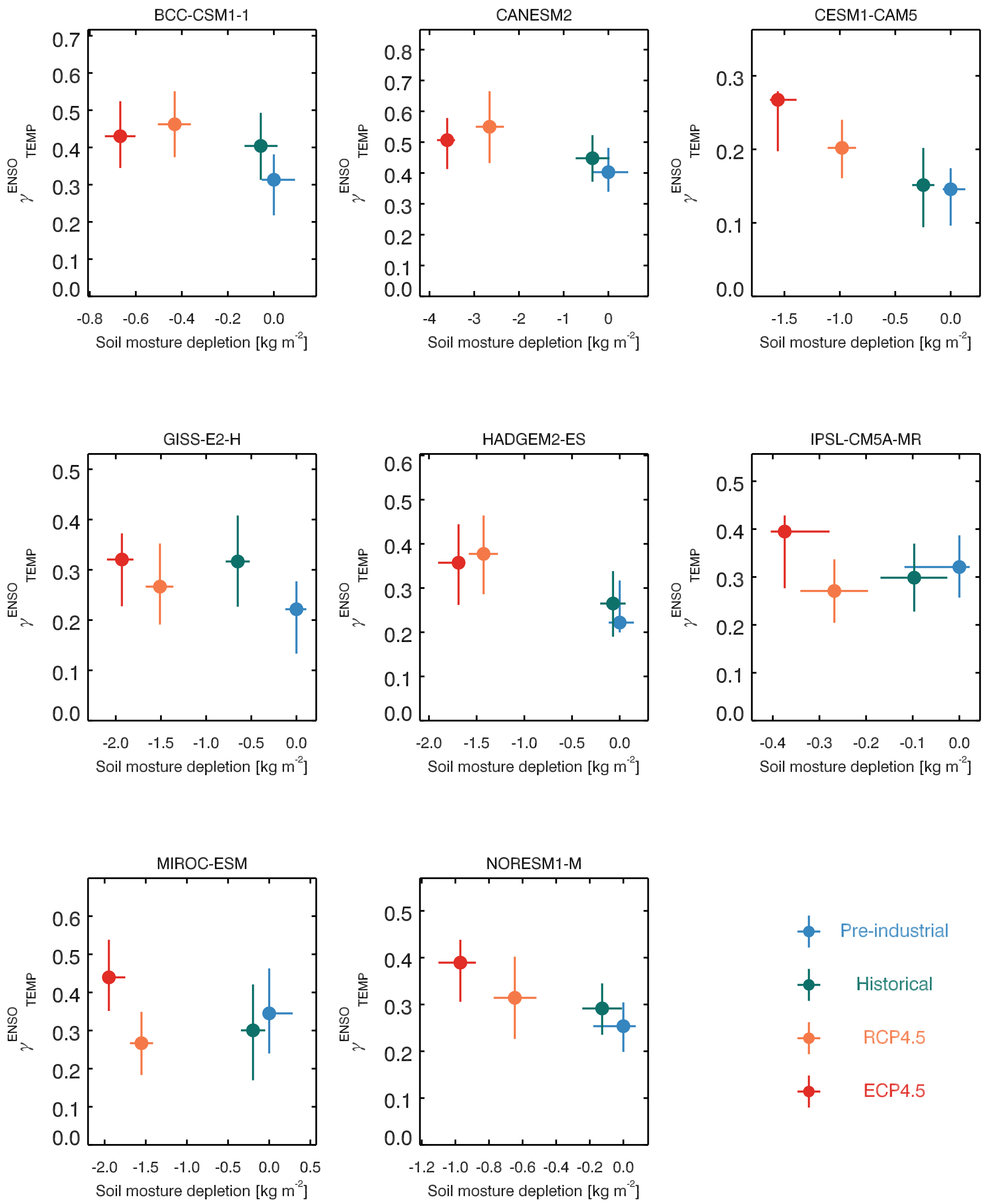

Supplementary Figure 11 | Soil moisture depletion and surface temperature anomalies related to ENSO in Amazonia. Mean soil moisture depletion during September to the following February (SONDJF) in Amazonia (100°–165°W, 20°S–10°N) for pre-industrial (blue), historical (green), RCP4.5 (orange) and ECP4.5 (red) and sensitivity of SONDJF mean surface temperature in Amazonia to ENSO ($\gamma^{\mathrm{ENSO}}_{\mathrm{Temp}}$) based on the linear regression against December–February Niño3.4 index. Lines indicates 95% confidence levels calculated using bootstrap method.
